# Impact of the *Apolipoprotein E* (epsilon) Genotype on Cardiometabolic Risk Markers and Responsiveness to Acute and Chronic Dietary Fat Manipulation

**DOI:** 10.3390/nu11092044

**Published:** 2019-09-01

**Authors:** Kumari M. Rathnayake, Michelle Weech, Kim G. Jackson, Julie A. Lovegrove

**Affiliations:** 1 Hugh Sinclair Unit of Human Nutrition, Department of Food & Nutritional Sciences and Institute for Cardiovascular and Metabolic Research and Institute for Food, Nutrition and Health, University of Reading, Whiteknights, P.O. Box 226, Reading RG6 6AP, UK; 2 Department of Applied Nutrition, Faculty of Livestock, Fisheries and Nutrition, Wayamba University of Sri Lanka, Makandura 60170, Sri Lanka

**Keywords:** *APOE*, cardiometabolic risk markers, dietary fat, fat manipulation

## Abstract

*Apolipoprotein* (*APO*) *E* (ε) genotype is considered to play an important role in lipid responses to dietary fat manipulation but the impact on novel cardiometabolic risk markers is unclear. To address this knowledge gap, we investigated the relationship between the *APOE* genotype and cardiometabolic risk markers in response to acute and chronic dietary fat intakes. Associations with fasting (baseline) outcome measures (*n* = 218) were determined using data from the chronic DIVAS (*n* = 191/195 adults at moderate cardiovascular disease risk) and acute DIVAS-2 (*n* = 27/32 postmenopausal women) studies examining the effects of diets/meals varying in saturated, polyunsaturated and monounsaturated (MUFA) fatty acid composition. Participants were retrospectively genotyped for *APOE* (rs429358, rs7412). For baseline cardiometabolic outcomes, *E4* carriers had higher fasting total and low-density lipoprotein-cholesterol (LDL-C), total cholesterol: high-density lipoprotein-cholesterol (HDL-C) and LDL-C: HDL-C ratios, but lower C-reactive protein (CRP) than *E3/E3* and *E2* carriers (*p* ≤ 0.003). Digital volume pulse stiffness index was higher in *E2* carriers than the *E3/E3* group (*p* = 0.011). Following chronic dietary fat intake, the significant diet × genotype interaction was found for fasting triacylglycerol (*p* = 0.010), with indication of a differential responsiveness to MUFA intake between the *E3/E3* and *E4* carriers (*p* = 0.006). Test fat × genotype interactions were observed for the incremental area under the curve for the postprandial apolipoprotein B (apoB; *p* = 0.022) and digital volume pulse reflection index (DVP-RI; *p* = 0.030) responses after the MUFA-rich meals, with a reduction in *E4* carriers and increase in the *E3/E3* group for the apoB response, but an increase in *E4* carriers and decrease in the *E3/E3* group for the DVP-RI response. In conclusion, baseline associations between the *APOE* genotype and fasting lipids and CRP confirm previous findings, although a novel interaction with digital volume pulse arterial stiffness was observed in the fasted state and differential postprandial apoB and DVP-RI responses after the MUFA-rich meals. The reported differential impact of the *APOE* genotype on cardiometabolic markers in the acute and chronic state requires confirmation.

## 1. Introduction

The *apolipoprotein (APO) E* (ε) genotype is the most widely researched single nucleotide polymorphism in relation to cardiovascular disease (CVD) risk, with the *APOE4* allele linked with increased total cholesterol (TC) and low-density lipoprotein cholesterol (LDL-C), CVD risk and mortality [1,2,3,4,5]. The *APOE* genotype has also been reported to influence the fasting lipid profile in response to dietary fat intake. Studies to date have focused on high-fat, high saturated fatty acid (SFA), low-fat and high-fat, high-SFA with fish oil interventions [6,7,8,9,10,11,12], whilst little is known of the interactions between the *APOE* genotype with *n*-6 polyunsaturated (PUFA) and monounsaturated (MUFA) fatty acid intakes. This is particularly important and timely given that population dietary recommendations for CVD prevention advise the reduction of SFA intakes to ≤10% of total energy (%TE) via replacement with *n*-6 PUFA or MUFA. Although fasting lipids contribute to the increased CVD risk they do not seem to solely explain this increased risk in *APOE4* carriers, with limited information available on other cardiometabolic risk markers. In particular, endothelial dysfunction is now recognised as a key modifiable event in coronary atherosclerosis, but limited data are available on the impact of *APOE* on the responsiveness of vascular reactivity to dietary fat composition [13].

Most of the studies investigating the interaction between the *APOE* genotype with dietary fat intake on lipid metabolism have been performed in the fasted state. However, postprandial lipaemia is now recognised as an independent CVD risk factor [14,15,16], which is particularly relevant given that individuals are in the fed state for the majority of the day. Previous studies have reported polymorphisms in the *APOE* gene to be associated with increased postprandial triacylglycerol (TAG) responses [17,18,19]. The Reading, Imperial, Surrey, Cambridge and Kings (RISCK) study reported differential effects on the lipid response when SFA was replaced with MUFA and low glycaemic index carbohydrates after a 24-week dietary intervention with variations in the *APOE* genotype [20]. However, data is extremely limited on the impact of meal fatty acids on postprandial lipid and vascular outcomes according to the *APOE* genotype. 

The present analysis explored the interaction of the *APOE* genotype with both chronic and acute intake of diets/meals rich in SFA, MUFA or *n*-6 PUFA on established and novel cardiometabolic risk markers. This was achieved using data form the chronic Dietary Intervention and Vascular Function (DIVAS) study performed in 195 individuals with moderate CVD risk [21], and the DIVAS-2 postprandial study conducted in 32 postmenopausal women. We hypothesised that the *APOE* genotype would influence these risk markers both at baseline and in response to fat manipulation. The overall diet/meal fat effects for both studies will not be the focus of the current manuscript as these data have been previously reported for each subject group [21,22].

## 2. Methods

### 2.1. Study Participants and Design

This paper was based on a retrospective *APOE* genotype analysis and previously analysed cardiometabolic risk markers in participants from two studies (DIVAS and DIVAS-2) performed at the Hugh Sinclair Unit of Human Nutrition (University of Reading, Reading, UK). The details of the chronic DIVAS and acute DIVAS-2 studies have been previously published [21,22]. Only participants who had provided informed consent for the retrospective genotyping for *APOE* were included in this data analysis (*n* = 191 out of 195 participants for DIVAS and *n* = 27 out of 32 for DIVAS-2). Furthermore, five women had participated in both the DIVAS and DIVAS-2 studies, so only baseline data from the DIVAS study for these participants were included. Both studies were conducted in accordance with the Declaration of Helsinki.

### 2.2. Baseline Associations Between APOE Genotype with Established and Novel Cardiometabolic Risk Markers 

Baseline data (*n* = 218) from both the DIVAS (*n* = 84 men and *n* = 107 women) [21] and DIVAS-2 (*n* = 27 postmenopausal women) [21,22] studies were combined to investigate the impact of *APOE* genotype on vascular function, blood pressure, biomarkers of endothelial dysfunction, lipids, glucose, insulin and inflammatory markers determined in the fasting state. Habitual dietary intake according to the *APOE* genotype was assessed using data extracted from 4-day weighed food diaries and analysed using Dietplan (DIVAS: version 6.6; DIVAS-2: version 7; Forestfield, Horsham, UK). 

### 2.3. Impact of the APOE Genotype on the Responsiveness of Cardiometabolic Risk Markers

#### 2.3.1. Chronic Dietary Fat Composition

The DIVAS study was a single-blind, parallel-group randomised controlled trial that replaced 9.5–9.6 %TE of dietary SFA with MUFA or *n*-6 PUFA for 16 weeks. Non-smoking women and men (*n* = 191) aged 21–60 y identified as having moderate CVD risk were recruited in three cohorts from November 2009 to June 2012 [23]. Participants were randomly assigned to follow one of three intervention diets, stratified by sex, age, body mass index (BMI) and CVD risk score. The isoenergetic diets (target compositions of total fat:SFA:MUFA:n-6 PUFA as %TE) were rich in SFA (36:17:11:4), MUFA (36:9:19:4) or *n*-6 PUFA (36:9:13:10), and were matched for protein, carbohydrate and *n*-3 PUFA [23]. A flexible food-exchange model replaced sources of exchangeable fats in the diet with intervention foods that had a specific fatty acid composition, which included oils, spreads, snacks, and dairy products. Primary sources of exchangeable fats were: Butter (SFA-rich diet; Wyke Farms, Somerset, UK); refined olive oil and MUFA-rich spread (MUFA-rich diet; Unilever R&D, Vlaardingen B.V, Netherlands); safflower oil and *n*-6 PUFA-rich spread (*n*-6 PUFA-rich diet; Unilever R&D Vlaardingen B.V, Netherlands). At baseline (week 0) and after the intervention period (week 16), macro-vascular reactivity was assessed by conducting flow mediated dilatation (FMD) of the brachial artery and a fasted blood sample was taken, as previously described [21]. This study was approved for conduct by the West Berkshire Local Research Ethics Committee (09/H0505/56) and the Research Ethics Committee at the University of Reading (project reference number 09/40), and registered as a clinical trial at www.clinicaltrials.gov (NCT01478958).

#### 2.3.2. Acute Meal Fat Composition

The DIVAS-2 study was an acute, double-blind, randomised, cross-over study conducted between June 2014 and September 2015. Postmenopausal women (*n* = 32) were randomly assigned to consume sequential mixed test meals (0 min, 50 g fat and 330 min, 30 g fat) that were rich in SFA, MUFA or *n*-6 PUFA on three different occasions that were 4–6 weeks apart. Test fats included butter (SFA meal), refined olive oil and MUFA-rich spread (MUFA meal), and safflower oil and *n*-6 PUFA-rich spreads (*n*-6 PUFA meal). Details of the test meal fat composition and study procedures are given elsewhere [22]. Blood samples were collected regularly (every 30 min until 180 min, followed by every 60 min until 300 min) after breakfast until the participant was provided with lunch at 330 min. Blood samples were then collected at 30 min intervals until 420 min, with the last sample being taken at 480 min. In this study, FMD was performed at baseline (fasting), 180, 300 and 420 min, and clinic blood pressure, laser Doppler imaging (LDI) with iontophoresis and digital volume pulse (DVP) at baseline, 240 and 450 min. This study was approved for conduct by the Research Ethics Committee at the University of Reading (project reference number 14/16) and was registered at www.clinicaltrials.gov (NCT02144454).

### 2.4. Vascular Reactivity Measurements and Blood Pressure

For both DIVAS and DIVAS-2 studies, macro- and micro-vascular reactivity were assessed by conducting FMD of the brachial artery (primary outcome measure) and LDI with iontophoresis, respectively [24]. In the peripheral arteries, DVP (Pulse Trace PCA2; Micro Medical Ltd., Chatham, UK) assessed arterial stiffness and vascular tone by measuring the stiffness index (DVP-SI; m/s) and reflection index (DVP-RI; %), respectively [24]. In DIVAS, 24 h ambulatory blood pressure (ABP) and heart rate measurements were taken at 30 min intervals throughout the day and 60 min intervals during the night at baseline and week 16 using A/A grade automated oscillometric ABP monitors (A & D Instruments Ltd., Abingdon, UK) as described elsewhere [21]. In DIVAS and DIVAS-2, clinic measurements of systolic blood pressure (SBP), diastolic blood pressure (DBP) and heart rate were recorded at each study visit using an OMRON blood pressure monitor (OMRON Healthcare UK Ltd., Milton Keynes, UK). The difference between the average systolic and diastolic blood pressures was used to determine pulse pressure.

### 2.5. Biochemical Analysis, Estimates of Insulin Sensitivity/Resistance and CVD Risk Score

Serum samples from both studies were used to determine lipids (TC, high density lipoprotein-cholesterol (HDL-C), TAG, apolipoprotein (apo)B (DIVAS-2 only)), glucose, C-reactive protein (CRP) and non-esterified fatty acids (NEFA) using an ILAB600 clinical autoanalyzer (reagents and analyser: Werfen, UK, Warrington UK.; NEFA reagent: Alpha Laboratories, Eastleigh, UK; apoB reagent: Randox Laboratories Ltd., Crumlin, UK). The Friedewald formula was used to estimate fasting concentrations of LDL-C [25]. The use of commercial ELISA kits determined concentrations of serum insulin (Dako UK Ltd.; Ely, UK), and plasma concentrations of soluble intercellular adhesion molecule-1 (sICAM-1), soluble vascular cell adhesion molecule (sVCAM-1), P-selectin and E-selectin (R & D Systems, Biotechne, Abingdon, UK). Plasma nitrite and nitrate levels were analysed with ozone-based chemiluminescence [26] in the DIVAS study and Eicom NOx Analyser ENO-30 (Eicom; San Diego, CA, USA) [27], which is a HPLC-based approach, was used in the DIVAS-2 study.

Using baseline measures, standard equations were used to calculate the homeostatic model assessment-insulin resistance (HOMA-IR) and the revised quantitative insulin sensitivity check index (rQUICKI) as measures of insulin resistance and insulin sensitivity, respectively [28]. Estimation of 10 y CVD risk was determined using the QRISK^®^2-2016 online risk calculator (https://qrisk.org/2017/).

### 2.6. DNA Extraction and Genotyping

The buffy coat was isolated from 9 mL of blood collected into K2EDTA blood collection tube, and DNA was extracted using the Qiagen DNA Blood Mini Kit (Qiagen Ltd., Crawley, UK). *APOE* genotype (*E2/E4*, *E2/E3*, *E2/E2, E3/E3, E3/E4* or *E4/E4*) was determined retrospectively by allelic discrimination using “Assay-on-Demand” single nucleotide polymorphism genotyping assays (rs7412 and rs429358; Life Technologies, Paisley, UK). 

### 2.7. Statistical Analysis

The DIVAS and DIVAS-2 studies were powered to detect a 2% (SD 2.3%, 80% power and 5% significance level) and 1.5% (SD 2.0%, 80% power and 5% significance level) difference in %FMD response (primary outcome), requiring 171 and 28 participants, respectively. Secondary outcome measures in both studies included arterial stiffness, microvascular reactivity, blood pressure, serum lipid profile, circulating markers of endothelial activation and inflammation and estimates of insulin sensitivity/resistance. The analysis presented in this manuscript is explorative, investigating the interactions between *APOE* genotype with fat manipulation on the primary and secondary outcome measures.

Data analysis was performed using the statistical analysis software SPSS, version 21 (SPSS Inc., Chicago, IL, USA). Results are presented in the text, tables and figure as means ± SEMs. *p* ≤ 0.05 was considered significant. Data were checked for normality of distribution, and skewed variables were normalized prior to statistical analysis. For the baseline data analysis, which included data from both the DIVAS and DIVAS-2 studies, a univariate general linear model (analysis of covariance—ANCOVA) was implemented using the baseline outcome measures as the dependent variables, with sex and the *APOE* genotype included as fixed factors and age and BMI as covariates, to assess the *APOE* genotype effect. If a significant genotype effect was observed, pairwise comparisons were carried out. These included a Bonferroni correction in which *p* ≤ 0.017 was considered significant.

To determine the effects of chronic dietary fat manipulation (DIVAS), a general linear model (ANCOVA) was used to determine the overall effect of diet and the *APOE* genotype on the primary and secondary outcome measures. In this model, the post-intervention (week 16)—baseline (week 0) difference was the dependent variable, with the genotype, sex and intervention diet as fixed factors, and baseline value of the variable of interest, age and BMI included as covariates. The interaction term was included in the model to assess the overall diet × *APOE* genotype interaction. If a significant interaction was found, a general linear model was performed for the three diets to determine which diets were different within each genotype group separately. When there was no overall diet effect for each genotype group, independent *t*-tests were performed for the three diet groups separately to identify whether there were any differences between the genotype groups.

Acute effects of test fat composition (DIVAS-2) on the time response profiles were analysed using a mixed factor repeated measures ANOVA with test fat and time included as within-subject factors and genotype as the between-group factor. Postprandial response summary measures were expressed as area and incremental area under the curve (AUC and IAUC, respectively) over 420, 450 or 480 min. The IAUC denotes the specific response to the test meals irrespective of baseline concentrations. For NEFA, AUC and IAUC were computed from the mean time of suppression until the final postprandial time point (120–480 min). Non-parametric one-way repeated measures ANOVA was used for the statistical analysis of IAUC with negative values and for any data that was not normalized following transformation. If a significant test fat × genotype interaction was found, a repeated measures ANOVA was performed in the two genotype groups separately, with a Bonferroni correction (where *p* ≤ 0.017 was considered significant). An independent *t*-test compared the responses to the different test fats between genotype groups, where values *p* ≤ 0.05 were significant. 

## 3. Results

### 3.1. Baseline Associations between the APOE Genotype with Established and Novel Cardiometabolic Risk Markers 

Table 1 presents the baseline subject characteristics and cardiometabolic risk markers of the 216 participants (84 males and 132 females (self-reported menopausal status: 66 pre-, 8 peri- and 58 postmenopausal women)) according to the *APOE* genotype, which were presented as *E2* carriers (*E2/E2* and *E2/E3*, *n* = 30), the wild-type homozygous *E3/E3* group (*n* = 128) and *E4* carriers (*E3/E4* and *E4/E4*, *n* = 58). Individuals with the *E2/E4* genotype were excluded from all data analyses due to the small subject group (*n* = 2).

At baseline, there was no significant effect of genotype on the %FMD response (primary outcome). For TC (*p* = 0.0001), LDL-C (*p* = 0.0001), TC:HDL-C ratio (*p* = 0.002) and LDL-C:HDL-C ratio (*p* = 0.0001), a significant genotype effect was evident with lipid concentrations and ratios increasing in the order: *E2* carriers > *E3/E3* group > *E4* carriers (Table 1). There was also an influence of genotype on baseline CRP (*p* = 0.002), with lower concentrations in *E4* carriers compared with the wild-type group (*p* = 0.003) and *E2* carriers *(p* = 0.002). DVP-SI was found to be different between genotype groups (*p* = 0.027), with a 17% higher DVP-SI in the *E2* carriers than the *E3/E3* group (*p* = 0.011). The *APOE* genotype did not influence any of the other baseline characteristics or cardiometabolic risk markers (Table 1). Habitual dietary intakes stratified according to the genotype (Table S1) showed differences in %TE of trans fatty acids (*p* = 0.031), whereby intakes were greater in the *E2* carriers than *E3/E3* group (*p* = 0.043). However, this difference was not significant after correcting for multiple comparisons (*p* ≥ 0.017).

### 3.2. Effect of Dietary Fat Manipulation and the APOE Genotype on Cardiometabolic Risk Markers

Since low numbers of *E2* carriers (*E2/E2* and *E2/E3*) were identified by retrospective genotyping, they were excluded from the datasets for the (i) chronic (*n* = 27, SFA diet (*n* = 12), MUFA diet (*n* = 5) and *n*-6 PUFA diet (*n* = 10)) and (ii) acute (*n* = 3, *E2/E3*) fat manipulations.

#### 3.2.1. Chronic Dietary Fat Composition (DIVAS)

In this analysis, a total of 159 subjects (*n* = 68 men and 91 women) were included with a mean age of 44 ± 1 y and mean BMI of 26.4 ± 0.3 kg/m^2^, of which 107 had the *E3/E3* genotype and 52 were *E4* carriers. *E2* carriers (*n* = 32) were excluded from this analysis due to relatively small numbers within each dietary intervention group (Table 2). No diet × genotype interaction was evident for the change in the primary outcome, %FMD response, or other measures of vascular function during the 16-wk chronic intervention. A significant diet × genotype interaction was found for the change in fasting TAG (*p* = 0.010) but there was no overall diet effect when the *APOE3/E3* and *E4* carrier groups were analysed separately. However, there was an indication of a differential responsiveness of fasting TAG to MUFA intake (but not SFA and *n*-6 PUFA) with an increase and decrease in TAG concentration in the *E3/E3* and *E4* carriers, respectively (*p* = 0.006; Table 2). *APOE* genotype was not found to influence any of the other secondary outcome measures in response to chronic dietary fat intake.

Independent of the 16-wk dietary intervention, genotype effects were observed for the changes from baseline for HDL-C (*p* = 0.015), CRP (*p* = 0.036), as well as P-selectin (*p* = 0.026; Table 2) where there was a reduction in HDL-C and CRP in the *E4* carriers as opposed to an increase in the *E3/E3* group, and an increase in P-selectin in the *E4* carriers relative to the decrease observed in the *E3/E3* group.

#### 3.2.2. Acute Meal Fat Composition (DIVAS-2)

**Figure 1:**
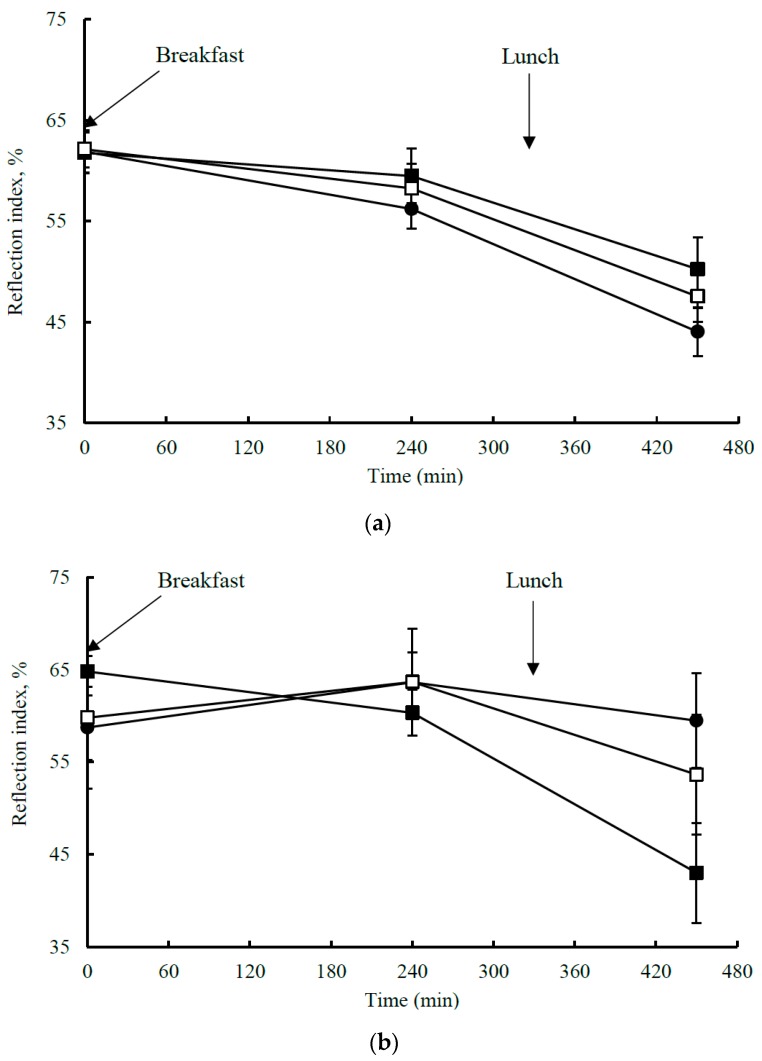
Mean ± SEM for the postprandial digital volume pulse reflection index (DVP-RI) response in (**a**) the *E3/E3* group (*n* = 22) and (**b**) *E4* carriers (*n* = 5) response following sequential meals (breakfast: 0 min and lunch: 330 min) enriched in saturated fatty acids (SFA; ■), monounsaturated fatty acids (MUFA; □) and *n*-6 polyunsaturated fatty acids (PUFA; ●). Two-way repeated measures ANOVA revealed a significant test fat × time × genotype interaction (*p* = 0.014) for the DVP-RI response. There was also a significant genotype × test fat interaction for the DVP-RI IAUC (*p* = 0.030).

**Figure 2:**
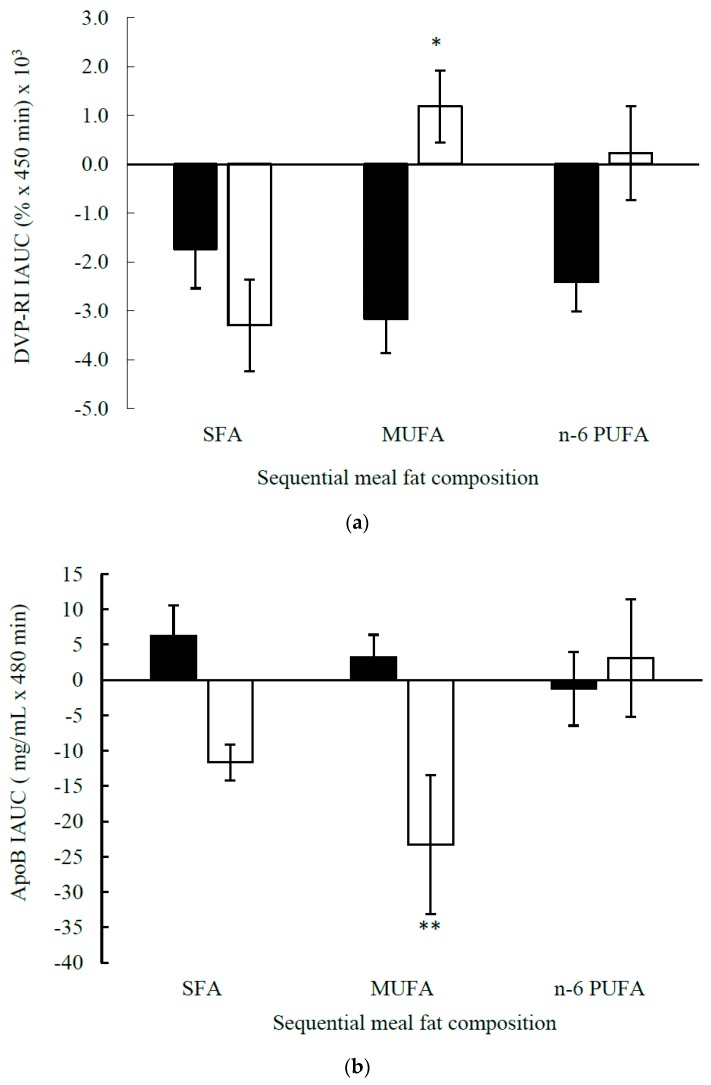
Incremental area under the curve (IAUC) for the postprandial (**a**) digital volume pulse reflection index (DVP-RI) and (**b**) total serum apolipoprotein (apo)B response according to *APOE* in the postmenopausal women following sequential meals (breakfast: 0 min and lunch: 330 min) enriched in saturated fatty acids (SFA), monounsaturated fatty acids (MUFA) and *n*-6 polyunsaturated fatty acids (PUFA). Data represent mean ± SEM for the *APOE3/E3* group (black bars, *n* = 17) and *APOE4* carriers (white bars, *n* = 4). There was a significant genotype × test fat interaction for the DVP-RI IAUC (*p* = 0.030) with an increase in the DVP-RI IAUC in *E4* carriers compared to a reduction in the *E3/E3* group (* *p* = 0.002) to the MUFA-rich meal. There was a significant genotype × test fat interaction for the postprandial total apoB IAUC (*p* = 0.022) with a reduction in the apoB IAUC in *E4* carriers compared to an increase in the *E3/E3* group after the MUFA-rich meals (** *p* = 0.002).

This analysis included 27 postmenopausal women (*n* = 22, *E3/E3* and *n* = 5, *E3/E4)*, with a mean age of 58 ± 1 y and mean BMI of 26.1 ± 0.7 kg/m^2^ (Table 3). The *APOE* genotype did not influence the responsiveness of postprandial measures of macrovascular function (%FMD response), microvascular function (LDI) or arterial stiffness (DVP-SI) to the meal fat composition. However, the postprandial DVP-RI time response profile showed a significant test fat × time × *APOE* genotype interaction (*p* = 0.014; Figure 1a,b). This was associated with a significant genotype × test fat interaction for the DVP-RI IAUC (*p* = 0.030; Figure 2a) with an increase in the DVP-RI IAUC in *E4* carriers as opposed to a reduction in the *E3/E3* group (*p* = 0.002) to the MUFA-rich meal. When data were split according to the genotype group, there was a significant test fat × time interaction (*p* = 0.037) and test fat effect (*p* = 0.027) in the *E4* carriers only, in which the SFA-rich meal reduced the DVP-RI IAUC relative to MUFA (*p* = 0.033) and *n*-6 PUFA-rich (*p* = 0.028) meals, although these effects were not considered significant after applying the Bonferroni correction (*p* ≥ 0.017).

There was a test fat × genotype interaction for the total serum postprandial apoB response IAUC (*p* = 0.022), with a tendency for higher IAUC after the *n*-6 PUFA than MUFA and SFA-rich meals (*p* = 0.068) in *E4* carriers only (Table 3, Figure 2b). However, there were differential effects on the responsiveness of the genotype groups to the MUFA-rich meals, with a reduction in the total apoB IAUC in *E4* carriers compared to an increase in the *E3/E3* group (*p* = 0.002). For all other cardiometabolic risk markers, the *APOE* genotype did not appear to influence the postprandial responses to the test fats.

## 4. Discussion

It has been suggested that personalised gene-based dietary advice is more useful than general dietary guidelines [29] and more effective at motivating dietary change [30]. It is, therefore, important to investigate common single nucleotide polymorphisms related with CVD risk that impact on response to key population dietary fat recommendations for CVD risk reduction. To our knowledge, this is the first data analysis that has examined both the chronic and acute impact of dietary fat manipulation on novel and established cardiometabolic risk markers according to the *APOE* genotype.

In the present baseline analysis, the higher fasting TC and LDL-C concentrations, and TC: HDL-C and LDL-C:HDL-C ratios were more evident in *E4* carriers than *E2* carriers and the *E3/E3* wild-type genotype group, which confirms previous studies [4,5,20,31,32,33]. A number of possible mechanisms could explain the higher fasting TC and LDL-C concentrations in *E4* carriers [11]. ApoE is present on TAG-rich lipoproteins (chylomicrons and very low-density lipoproteins (VLDL)) and HDL, but not LDL particles and is involved with lipid transport and receptor mediated clearance. The apoE4 protein isoform has selective affinity for larger TAG-rich lipoproteins, for example dietary derived chylomicrons, which would be expected to increase the competition with LDL for the LDL receptor mediated clearance, increasing circulating LDL-C concentrations [34,35]. However, the lower binding affinity of the *E2* isoform to the hepatic LDL receptor, compared with both *E3* and *E4* would be expected to slow the clearance of VLDL and dietary chylomicron remnants, and increase LDL clearance leading to typically lower concentrations of TC and LDL-C, yet higher postprandial TAG in *E2* carriers [36]. Furthermore, the lipolytic conversion of VLDL remnants to LDL is reportedly faster in *E4* carriers [37]. All of these mechanisms could contribute to varying extents to the higher fasted TC and LDL-C concentrations in *E4* carriers in the current study, in the order of *E4* carriers *> E3/E3* group *> E2* carriers.

We also observed genotype effects on a biomarker of inflammation, with *E2* carriers having greater fasting CRP concentrations compared with *E4* carriers. Additionally, serum CRP was lower in *E4* carriers, a finding that has also been observed in other studies [4,38,39,40,41,42,43,44,45]. Although both fasting LDL-C and CRP are recognised as independent CVD risk factors [46], some recent literature has indicated that elevated CRP does not raise the risk of CVD events as much as originally thought [47,48,49]. Therefore, the greater CVD risk previously reported in E4 carriers could be due to increased TC and LDL-C concentrations despite lower CRP [3,5]. Findings from an experimental study have reported apoE to influence immune cell function, with *APOE4* carriers more predisposed to a pro-inflammatory phenotype [50]. Further studies incorporating markers of immune function are needed to confirm these findings and determine the mechanisms linking the *APOE* genotype with inflammation.

The present study found a limited impact of the *APOE* genotype on the responsiveness of cardiometabolic risk markers to differences in chronic fat intake, although there was some evidence that *E4* carriers were more sensitive to the TAG lowering effect of a diet in which 8 %TE SFA was replaced with MUFA. Analysis of data from the Diet, genomics and metabolic syndrome: an integrated nutrition, agro-food, social and economic analysis (LIPGENE; *n* = 442 men and women classified with metabolic syndrome) and RISCK (*n* = 389 men and women ‘at risk’ from metabolic syndrome) studies revealed no differences in fasted TAG following a 19 and 17 %TE MUFA diet for 12 or 16 weeks, respectively, according to the *APOE* genotype [33]. However, analysis of the data from the LIPGENE study was performed according to plasma fatty acids concentrations, rather than dietary intake, and although circulating fatty acids (particularly PUFA) can reflect consumption, only weak correlations between dietary and plasma SFA and MUFA exist due to endogenous de novo synthesis of these fatty acids from lipid and non-lipid sources [51]. Furthermore, a study in 84 young healthy students with a mean age of 22.5 years (66 *APOE3/3*, 8 *APOE4/E3* and 10 *APOE2/E3*) following a 22 %TE MUFA diet for 4 weeks also reported no difference in TAG according to the *APOE* genotype, although there were low numbers of *E4* carriers and could have been underpowered for these comparisons as a result of retrospective genotyping [52]. Additional research is required to confirm the effects of different unsaturated fats on fasting TAG according to *APOE* genotype.

Our study found the *APOE* genotype had a limited impact on postprandial lipid levels in relation to dietary fat manipulation, which is in agreement with our SATurated fat and gene *APOE* (SATgene) study [7], which prospectively genotyped according to *APOE* (*E3/E3* and *E4* carriers). However, in the current study a greater reduction in the postprandial apoB response (IAUC) was identified in *E4* carriers compared with the *E3/3* group after the MUFA-rich meals, although no difference in TAG responses were evident. This was surprising since TAG is transported in chylomicrons (containing apoB48) and VLDL (containing apoB100) and postprandial total apoB generally reflects concentrations of these TAG-rich lipoproteins. In contrast, Cardona et al. investigated adults identified as having the metabolic syndrome and found no impact on apoB measured 4 h postprandially after a MUFA-rich meal, but observed *E2* and *E4* carriers combined to have higher postprandial TAG, with *E2* carriers having the highest TAG [19]. Postprandial differences in TAG were observed after a SFA-rich test meal in *E4* carriers compared with *E3/E3*, although these were reported to be reflective of the higher baseline TAG concentrations [18]. In support of the importance of fasted TAG concentrations, the significantly higher postprandial TAG concentrations in *E4* carriers compared with *E3/E3* in response to a high fat meal were attenuated when the incremental response was calculated [17].

We found no differences in measures of fasted macrovascular (%FMD response) or microvascular (LDI) reactivity, however we provided novel evidence of a difference in baseline (fasting) arterial stiffness according to the *APOE* genotype. Vascular dysfunction is considered to be an important risk factor for CVD and associations have been reported between cardiovascular mortality and related vascular conditions, including hypertension [53], arterial stiffness [54] and endothelial dependent vasodilation [55], and arterial stiffness is related to chronic inflammation and dyslipidaemia, particularly elevated TAG [56], often found in *E2* carriers, with *E2* homozygotes having a higher risk of Type III hyperlipoproteinemia, which leads to progressive atherosclerosis [35,38]. The higher arterial stiffness (measured by DVP-SI) observed in the *E2* carriers, compared with *E3/E3* in our study, might have reflected the higher inflammatory marker (CRP) in our *E2* carriers, although further confirmation of this association is required. However, there was no diet/meal fat and *APOE* genotype interaction observed for arterial stiffness. This is perhaps not surprising as arterial stiffness is a progressively slow process and longer term dietary fat manipulation may be necessary before differences are observed [57]. With regards to vascular function, this study is the first to report that the *APOE* genotype had a limited impact on fasting and postprandial measures of vascular function after chronic and acute fat manipulation, respectively. Yet, a reduction in DVP-RI was observed in the *E3/E3* compared with *E4* carriers after the MUFA-rich meals, which indicates that differences in small vessel tone after meal ingestion are dependent on genotype, although the mechanism of action and the clinical relevance are unclear.

This study is novel, being the first to investigate the effect of the *APOE* genotype on vascular function and cardiometabolic risk markers at baseline and in response to chronic and acute dietary fat manipulation. Furthermore, there are a number of strengths in the study design of both the chronic and acute interventions. Firstly, the target intakes of SFA in the unsaturated fat diets in the chronic DIVAS study were compliant with the current population recommendation for CVD risk reduction of ≤10 %TE SFA. Secondly, the chronic diets were followed for a longer duration (16 weeks) than many published dietary fat interventions investigating the effects on vascular function as the primary outcome. Moreover, the acute study (DIVAS-2) used a two meal sequential postprandial protocol, which is considered superior to a single test meal challenge as it better represents a habitual meal intake pattern [58,59]. Since the genotyping was performed retrospectively, the number of participants who carried the *E2* allele was low in both the chronic and acute datasets, which necessitated removal of *E2* carriers, and could be considered as a limitation. Furthermore, there was a small sample size for *E4* carriers in the DIVAS-2 study. In addition, as only postmenopausal women were recruited for the postprandial analysis (DIVAS-2), our postprandial findings may not relate to other population subgroups, including men and premenopausal women. However, both the baseline and chronic data analyses included a wider population, consisting of both men and pre- and postmenopausal women in the UK.

In conclusion, this study has confirmed previous findings that the *APOE* genotype is associated with fasting lipid profile and CRP and presents novel evidence of an association between the *APOE* genotype with fasting DVP-SI. Moreover, our findings revealed a limited influence of the *APOE* genotype on the responsiveness of novel and established cardiometabolic risk markers to chronic and acute fat manipulation. However, further studies are warranted using prospective genotyping in relation to dietary fat recommendations for CVD risk reduction to confirm the findings in relation to the effects of the *APOE* genotype on markers of vascular functions, lipids and inflammation.

## Figures and Tables

**Table 1:** Baseline characteristics in the combined study group and according to the *APOE* genotype.

	All (*n* = 216)	*E2* Carriers (*n* = 30)	*E3/E3* (*n* = 128)	*E4* Carriers (*n* = 58)	*P* (Genotype) ^1^
**Genotype Frequency (%)**	-	14	59	27	
**Characteristics**					
Sex, M/F	84/132	13/17	43/85	28/30	
Age, y	46 ± 1	48 ± 2	45 ± 1	46 ± 2	0.537
Weight, kg	76.5 ± 1.0	78.2 ± 2.8	75.0 ± 1.2	78.9 ± 1.8	0.082
BMI, kg/m^2^	26.6 ± 0.3	27.7 ± 0.8	26.4 ± 0.3	26.5 ± 0.5	0.341
Waist circumference, cm	91.3 ± 0.8	96.2 ± 2.7	90.1 ± 1.0	91.3 ± 1.5	0.091
Waist:hip ratio	0.87 ± 0.01	0.90 ± 0.02	0.86 ± 0.01	0.88 ± 0.01	0.152
Clinic blood pressure					
Systolic, mm Hg	119 ± 1	122 ± 3	119 ± 1	118 ± 2	0.432
Diastolic, mm Hg	74 ± 1	77 ± 1	74 ± 1	73 ± 1	0.193
Pulse pressure, mm Hg	45 ± 1	45 ± 2	45 ± 1	45 ± 1	0.873
**Biochemical profile and CVD risk**					
TC, mmol/L	5.49 ± 0.07	4.77 ± 0.20 ^a^	5.49 ± 0.09 ^b^	5.88 ± 0.13 ^c^	0.0001
HDL-C, mmol/L	1.49 ± 0.02	1.43 ± 0.06	1.52 ± 0.03	1.47 ± 0.05	0.606
LDL-C, mmol/L	3.42 ± 0.06	2.73 ± 0.16 ^a^	3.42 ± 0.07 ^b^	3.77 ± 0.11 ^c^	0.0001
TC: HDL-C ratio	3.84 ± 0.07	3.49 ± 0.19 ^a^	3.77 ± 0.09 ^b^	4.20 ± 0.16 ^c^	0.002
LDL-C: HDL-C ratio	2.41 ± 0.06	2.02 ± 0.15 ^a^	2.37 ± 0.07 ^b^	2.72 ± 0.12 ^c^	0.0001
TAG, mmol/L	1.27 ± 0.04	1.34 ± 0.13	1.21 ± 0.05	1.34 ± 0.10	0.551
NEFA, µmol/L	502 ± 12	525 ± 28	509 ± 17	472 ± 22	0.413
Glucose, mmol/L	5.09 ± 0.03	5.12 ± 0.07	5.05 ± 0.04	5.18 ± 0.07	0.527
Insulin, pmol/L	31.2 ± 1.3	36.4 ± 4.6	30.4 ± 1.6	30.2 ± 2.3	0.619
HOMA-IR	1.19 ± 0.05	1.41 ± 0.19	1.14 ± 0.06	1.18 ± 0.10	0.605
rQUICKI	0.45 ± 0.01	0.43 ± 0.01	0.45 ± 0.01	0.46 ± 0.01	0.238
QRISK^®^2, ^2^ %	2.8 ± 0.2	3.3 ± 0.7	2.4 ± 0.2	3.3 ± 0.4	0.142
**Vascular function**					
%FMD response	6.2 ± 0.2	5.8 ± 0.5	6.4 ± 0.3	6.0 ± 0.4	0.698
LDI-Ach, AUC, PU	1548 ± 59	1529 ± 164	1523 ± 77	1601 ± 114	0.588
LDI-SNP, AUC, PU	1464 ± 50	1327 ± 106	1448 ± 64	1557 ± 105	0.370
DVP-RI, %	63.2 ± 0.9	64.8 ± 2.5	62.1 ± 1.1	64.8 ± 1.7	0.649
DVP-SI, m/s	6.9 ± 0.1	7.7 ± 0.5 ^a^	6.6 ± 0.1 ^b^	7.2 ± 0.2 ^a,b^	0.027
**Biomarkers of inflammation and endothelial activation**					
C-reactive protein, mg/L	2.23 ± 0.23	3.20 ± 0.70 ^a^	2.27 ± 0.29 ^a^	1.66 ± 0.41 ^b^	0.002
sVCAM-1, ng/mL	661 ± 11	653 ± 23	652 ± 16	685 ± 20	0.400
sICAM-1, ng/mL	218 ± 3	228 ± 9	220 ± 4	207 ± 5	0.120
E-selectin, ng/mL	34.2 ± 1.0	31.4 ± 2.5	34.5 ± 1.3	35.0 ± 1.9	0.189
P-selectin, ng/mL	40.7 ± 1.0	37.3 ± 2.5	41.0 ± 1.3	41.8 ± 1.9	0.078

Values represent mean ± SEM, *E2* carriers = *E2/E2* and *E2/E3*; *E4* carriers = *E3/E4* and *E4/E4*. *E2/E4* individuals were excluded from the analysis. ^1^ Data analysed by univariate general linear model (analysis of covariance—ANCOVA) adjusted for age, BMI and sex. If significant, pairwise comparisons were used to determine differences between genotype groups. ^2^ QRISK®2 10 y risk of cardiovascular disease (https://qrisk.org/2017/) ^a, b, c^ Different superscript letters within a row indicate significant differences between genotype groups (*p* ≤ 0.017). Abbreviations: Ach, acetylcholine; PU, perfusion units; DBP, diastolic blood pressure; DVP-RI, digital volume pulse reflection index; DVP-SI, digital volume pulse stiffness index; FMD, flow-mediated dilatation; HDL-C, high-density lipoprotein cholesterol; HOMA-IR, quantitative insulin resistance index; LDI, laser Doppler imaging; AUC, area under the curve; LDL-C, low-density lipoprotein cholesterol; NEFA, non-esterified fatty acids; rQUICKI, revised quantitative insulin sensitivity index; SBP, systolic blood pressure; sICAM-1, soluble intercellular adhesion molecule-1; SNP, sodium nitroprusside; sVCAM-1, soluble vascular cell adhesion molecule-1; TAG, triacylglycerol; TC, total cholesterol.

**Table 2:** Changes in fasting cardiometabolic risk markers after chronic dietary fat manipulation according to the *APOE* genotype (DIVAS study).

	*E3/E3* (*n* = 107)			*E4* Carriers (*E3/E4* and *E4/E4*, *n* = 52)				*p* Value ^1^
	SFA	MUFA	*n*-6 PUFA	SFA	MUFA	*n*-6 PUFA	Genotype	Diet × Genotype
*N*	35	36	36	17	17	18		
Age, y	44 ± 1	42 ± 2	43 ± 2	44 ± 3	46 ± 3	47 ± 3		
BMI, kg/m^2^	25.8 ± 0.8	26.5 ± 0.8	26.9 ± 0.6	27.5 ± 0.9	26.1 ± 1.0	25.9 ± 0.8		
**Biochemical profile and estimates of insulin sensitivity/resistance**								
TC, mmol/L	0.42 ± 0.10	−0.03 ± 0.13	−0.01 ± 0.13	0.21 ± 0.19	−0.29 ± 0.17	−0.19 ± 0.20	0.165	0.760
HDL-C, mmol/L	0.06 ± 0.03	0.04 ± 0.03	0.12 ± 0.04	0.03 ± 0.05	−0.05 ± 0.05	−0.02 ± 0.07	0.015	0.473
LDL-C, mmol/L	0.35 ± 0.09	−0.09 ± 0.11	−0.11 ± 0.10	0.17 ± 0.15	−0.19 ± 0.15	−0.18 ± 0.14	0.401	0.984
TC: HDL-C ratio	0.20 ± 0.08	−0.11 ± 0.07	−0.25 ± 0.07	0.01 ± 0.13	−0.13 ± 0.17	−0.05 ± 0.08	0.703	0.263
LDL-C: HDL-C ratio	0.19 ± 0.08	−0.12 ± 0.07	−0.23 ± 0.07	0.02 ± 0.11	−0.10 ± 0.15	−0.07 ± 0.08	0.652	0.324
TAG, mmol/L	−0.00 ± 0.05	0.10 ± 0.06 ^a^	−0.07 ± 0.05	0.06 ± 0.14	−0.23 ± 0.10 ^b^	0.08 ± 0.18	0.160	0.010
NEFA, µmol/L	−17.6 ± 35.1	−13.6 ± 22.1	−11.1 ± 21.5	−64.8 ± 40.2	46.5 ± 66.3	87.7 ± 26.2	0.413	0.082
Glucose, mmol/L	0.04 ± 0.05	0.07 ± 0.04	0.10 ± 0.06	0.08 ± 0.09	0.01 ± 0.06	0.01 ± 0.11	0.957	0.614
Insulin, pmol/L	1.10 ± 2.73	1.23 ± 1.86	2.67 ± 2.31	0.97 ± 2.77	0.49 ± 1.55	0.71 ± 1.67	0.851	0.857
HOMA-IR	0.07 ± 0.11	0.05 ± 0.08	0.12 ± 0.10	0.05 ± 0.12	0.03 ± 0.07	0.06 ± 0.07	0.821	0.930
rQUICKI	0.00 ± 0.01	−0.00 ± 0.01	−0.01 ± 0.01	0.00 ± 0.01	−0.01 ± 0.01	−0.02 ± 0.01	0.620	0.420
**Vascular function**								
%FMD response	−0.55 ± 0.33	0.33 ± 0.44	−0.09 ± 0.36	−0.40 ± 0.49	−0.20 ± 0.69	−0.95 ± 0.73	0.918	0.368
LDI ^2^								
LDI-Ach AUC, PU	−460.3 ± 198.1	−2.1 ± 154.6	91.1 ± 119.9	38.5 ± 164.6	−40.7 ± 232.8	−115.4 ± 142.2	0.438	0.134
LDI-SNP AUC, PU	−283 ± 173	−187 ± 158	131 ± 127	−28 ± 212	−296 ± 287	187 ± 174	0.233	0.601
DVP-RI, %	−1.98 ± 2.34	4.33 ± 2.57	4.29 ± 2.27	−0.27 ± 2.57	1.47 ± 1.91	−4.92 ± 3.52	0.078	0.171
DVP-SI, m/s	0.23 ± 0.32	0.76 ± 0.31	0.22 ± 0.37	0.37 ± 0.37	−0.15 ± 0.32	−0.93 ± 0.43	0.125	0.562
**Ambulatory blood pressure ^3^**								
24-h blood pressure								
SBP, mm Hg	1.7 ± 1.3	−1.1 ± 1.3	0.2 ± 1.8	0.5 ± 2.7	−0.9 ± 2.4	−1.3 ± 2.0	0.681	0.860
DBP, mm Hg	1.6 ± 0.9	−0.4 ± 0.9	−0.3 ± 1.1	1.1 ± 1.9	0.9 ± 1.7	−1.4 ± 1.3	0.921	0.813
Pulse pressure, mm Hg	0.2 ± 1.4	−0.7 ± 0.8	0.6 ± 1.0	−0.6 ± 1.1	−1.7 ± 1.4	0.1 ± 1.5	0.502	0.974
Heart rate, bpm	0.8 ± 1.0	0.7 ± 1.1	0.6 ± 1.1	2.8 ± 1.3	1.9 ± 1.8	−1.6 ± 1.6	0.565	0.292
**Biomarkers of inflammation and endothelial activation**								
C-reactive protein, mg/L	0.60 ± 0.60	0.04 ± 0.28	0.024 ± 0.51	−0.14 ± 1.14	−0.25 ± 0.57	−0.79 ± 0.63	0.036	0.786
NOx, µmol/L	0.51 ± 3.22	−1.89 ± 1.52	−1.78 ± 1.87	1.70 ± 3.48	4.43 ± 2.97	−2.27 ± 1.62	0.208	0.073
sVCAM-1, ng/mL	−40.0 ± 16.8	11.4 ± 22.2	2.3 ± 13.6	−2.1 ± 16.2	3.9 ± 35.4	25.1 ± 25.2	0.063	0.451
sICAM-1, ng/mL	−1.4 ± 4.2	1.3 ± 3.6	1.5 ± 6.3	5.2 ± 6.6	15.3 ± 8.2	12.8 ± 5.1	0.100	0.836
E-selectin, ng/mL	0.41 ± 1.39	−2.90 ± 1.15	−0.63 ± 1.03	0.70 ± 2.01	−3.03 ± 1.86	−0.24 ± 1.43	0.827	0.881
P-selectin, ng/mL	−0.94 ± 1.70	−1.94 ± 1.06	−3.49 ± 1.23	3.63 ± 1.74	−1.18 ± 2.32	0.21 ± 1.44	0.026	0.403

Values represent mean ± SEM, change from baseline after post intervention (week 16). Total *n* = 131–157, with *n* = 89–107 *E3/E3* and *n* = 4252 *E4* carriers per outcome. *E2*/*E4* and *E2* = *E2*/*E2* + *E2*/*E3* individuals were excluded from the analysis. ^1^ Data analysed by univariate general linear model (ANCOVA) by using the difference from baseline (post-intervention (Visit 2) minus baseline (Visit 1)) as the dependent variable, with genotype, sex and intervention diet as fixed factors and with baseline data for the variable of interest, age and BMI as covariates. The interaction term was added to the model to assess the *APOE* genotype and diet interaction. ^a,b^ Different superscript letters within a row indicate significant differences between genotype groups (*p* ≤ 0.017). ^2^ LDI-Ach and LDI-SNP were expressed as area under the curve (AUC) for the 20-scan protocol. Incremental AUC (IAUC) was also determined for the 20-scan protocol but differences between test fats for subsequent AUC and IAUC were not significant (data not shown). ^3^ Day and night ambulatory blood pressure were analysed and no significant effects were found (data not shown). Abbreviations: Ach, acetylcholine; PU, perfusion units; AUC, area under the curve, BMI, body mass index; DBP, diastolic blood pressure; DVP-RI, digital volume pulse reflection index; DVP-SI, digital volume pulse stiffness index; DIVAS, Dietary Intervention and vascular function; FMD, flow mediated dilatation; HDL-C, high-density lipoprotein cholesterol; HOMA, quantitative insulin resistance index; LDI, laser Doppler imaging; LDL-C, low-density lipoprotein cholesterol; MUFA, monounsaturated fatty acids; NEFA, non-esterified fatty acid; NOx, total nitrites and nitrates; PUFA, polyunsaturated fatty acids; rQUICKI, revised quantitative insulin sensitivity index; SBP, systolic blood pressure; SFA, saturated fatty acids; sICAM-1, soluble intercellular cell adhesion molecule 1; SNP, sodium nitroprusside; sVCAM-1, soluble vascular cell adhesion molecule 1; TAG, triacylglycerol; TC, total cholesterol.

**Table 3:** Summary measures for the impact of the *APOE* genotype on postprandial cardiometabolic risk markers after sequential meals of varying fat composition (DIVAS-2 study).

	*E3/E3* (*n* = 22)			*E3/E4* (*n* = 5)			*p* Value ^1^	
	SFA	MUFA	*n*-6 PUFA	SFA	MUFA	*n*-6 PUFA	Genotype	Test Fat × Genotype
**Biochemical measures ^2^**								
TAG, mmol/L								
AUC	942 ± 90	1022 ± 87	1035 ± 124	1063 ± 186	1022 ± 164	1125 ± 299	0.702	0.683
IAUC	320 ± 52	360 ± 53	361 ± 77	402 ± 94	403 ± 126	386 ± 115	0.702	0.828
NEFA, μmol/L								
AUC	143.5 ± 8.4	136.1 ± 11.3	126.9 ± 7.3	108.6 ± 16.1	102.4 ± 13.3	116.9 ± 21.1	0.147	0.382
IAUC	−73.5 ± 12.3	−87.9 ± 12.7	−83.7 ± 12.7	−75.2 ± 29.6	−12.6 ± 16.9	−62.0 ± 29.8	0.802	0.111
Apo B, mg/mL								
AUC	473 ± 18	469 ± 22	480 ± 25	549 ± 32	561 ± 50	512 ± 34	0.329	0.134
IAUC	62.4 ± 4.3	50.7 ± 3.2	−12.4 ± 5.2	−11.6 ± 2.5	−23.3 ± 9.8	31.3 ± 8.3	0.197	0.022 **^3^**
Glucose, mmol/L								
AUC	942 ± 90	1022 ± 87	1035 ± 124	1063 ± 186	1022 ± 164	1125 ± 299	0.666	0.299
IAUC	320 ± 52	360 ± 53	361 ± 77	402 ± 94	403 ± 126	386 ± 115	0.342	0.523
Insulin, μmol/L								
AUC	913 ± 8	877 ± 11	821 ± 9	948 ± 19	951 ± 15	952 ± 18	0.640	0.814
IAUC	881 ± 8	831 ± 9	779 ± 6	795 ± 16	783 ± 12	753 ± 14	0.610	0.750
**Vascular function ^2^**								
% FMD response								
AUC	1939 ± 148	2305 ± 191	2131 ± 191	2105 ± 235	2629 ± 486	2329 ± 218	0.510	0.898
IAUC	−149 ± 183	156 ± 261	114 ± 189	345 ± 335	431 ± 279	79 ± 281	0.433	0.683
LDI-Ach ^4^, AU × 10^3^								
AUC	742 ± 47	766 ± 56	772 ± 50	871 ± 101	973 ± 320	941 ± 160	0.888	0.931
IAUC	7.8 ± 35.1	−8.1 ± 62.2	−11.0 ± 53.9	−46.4 ± 101.6	−70.0 ± 132.3	−21.8 ± 127.9	0.088	0.975
LDI-SNP ^4^, AU × 10^3^								
AUC	755 ± 57	807 ± 66	652 ± 33	802 ± 99	957 ± 264	113 ± 227	0.690	0.083
IAUC	318 ± 60	−101 ± 59	−331 ± 61	−118 ± 74	−286 ± 117	−505 ± 171	0.754	0.624
DVP-RI, % × 10^3^								
AUC	26.1 ± 1.0	24.7 ± 0.7	25.5 ± 0.9	25.9 ± 0.8	27.6 ± 1.5	27.1 ± 2.7	0.407	0.392
IAUC	−1.7 ± 0.8	−3.2 ± 0.7	−2.4 ± 0.6	−3.3 ± 0.9	1.2 ± 0.7	0.2 ± 1.0	0.055	0.030 **^3^**
DVP-SI, m/s								
AUC	3193 ± 121	3074 ± 110	3169 ± 114	3463 ± 178	3505 ± 277	3617 ± 415	0.177	0.735
IAUC	14.6 ± 97.6	−181.2 ± 110.1	40.5 ± 120.4	171.3 ± 178.4	−44.0 ± 241.3	226.5 ± 154.4	0.373	0.987
SBP, mmHg × 10^3^								
AUC	56.3 ± 1.5	57.1 ± 1.4	57.1 ± 1.6	58.9 ± 3.5	56.7 ± 2.5	58.3 ± 3.5	0.716	0.267
IAUC	−2.5 ± 0.6	−4.8 ± 0.6	−3.3 ± 0.7	−3.6 ± 0.7	−3.2 ± 1.5	−4.2 ± 1.0	0.914	0.178
DBP, mmHg × 10^3^								
AUC	32.9 ± 3.3	33.1 ± 3.3	33.3 ± 3.3	32.9 ± 1.2	31.9 ± 1.0	32.0 ± 1.4	0.686	0.341
IAUC	−1.1 ± 0.4	−2.2 ± 0.3	−1.5 ± 0.3	−1.0 ± 0.6	−1.7 ± 0.3	−9.7 ± 0.7	0.442	0.867
**Biomarkers of endothelial activation ^2^**								
NOx, μmol/L								
AUC	5820 ± 482	5962 ± 708	5119 ± 389	6308 ± 258	6278 ± 258	6084 ± 1330	0.288	0.996
IAUC	−1618 ± 231	−1446 ± 370	−1057 ± 219	−2165 ± 521	−3015 ± 695	−1762 ± 393	0.078	0.318
sVCAM-1, µg/mL								
AUC	263.0 ± 7.1	270.7 ± 9.2	256.9 ± 7.3	263.4 ± 22.2	243.0 ± 14.6	243.5 ± 16.4	0.431	0.118
IAUC	4.2 ± 5.0	3.1 ± 4.6	−4.7 ± 2.9	3.9 ± 4.9	−8.5 ± 9.2	−9.6 ± 8.1	0.360	0.707
sICAM-1, µg/mL								
AUC	86.3 ± 3.1	876.9 ± 3.8	702.5 ± 5.7	715.3 ± 3.5	717.9 ± 2.9	588.9 ± 6.1	0.141	0.775
IAUC	−3.2 ± 1.3	−0.2 ± 1.5	−18.1 ± 4.1	0.4 ± 6.4	0.6 ± 2.5	−15.9 ± 10.0	0.581	0.881
E-selectin, µg/mL								
AUC	10.7 ± 0.9	11.0 ± 0.9	10.6 ± 0.8	11.4 ± 1.6	11.9 ± 1.5	10.6 ± 1.6	0.694	0.407
IAUC	−0.4 ± 0.3	−0.01 ± 0.2	−0.2 ± 0.1	−0.3 ± 0.3	0.6 ± 0.4	0.5 ± 0.01	0.065	0.472
P-selectin, µg/mL								
AUC	13.0 ± 0.7	13.0 ± 0.9	13.1 ± 0.8	14.7 ± 1.8	16.5 ± 2.9	14.8 ± 2.1	0.300	0.131
IAUC	−0.6 ± 0.3	0.01 ± 0.2	−0.01 ± 0.3	−0.07 ± 0.0	1.3 ± 1.3	0.3 ± 0.4	0.078	0.737

Values are mean ± SEM, for the *E3/E3* and *E3/E4* groups. *E2* carriers and *E2/E4* individuals were excluded from the analysis. ^1^ *p* value refers to the interaction between summary measures and the *APOE* genotype. A mixed factor repeated measures ANOVA determined the effects of the test fats on summary measures. For this, test fat and time were included as within-subject factors and genotype as the between group factor. ^2^ Units for AUC and IAUC expressed as biomarker units × time interval. The time interval for AUC and IAUC represents 480 min for TAG, apoB, glucose and insulin; 120–480 min for NEFA; 420 min for FMD and biomarkers of endothelial activation; 450 min for DBP, SBP, DVP-SI, DVP-RI and LDI. ^3^ For significant test fat × genotype interactions, independent samples *t*-test was performed to identify the effects of genotype for each test fat separately. ^4^ LDI-Ach and LDI-SNP were expressed as AUC for the 20-scan protocol. IAUC was also determined for the 20-scan protocol but differences between test fats for subsequent AUC and IAUC were not significant (data not shown). Abbreviations: Ach, acetylcholine; apoB, apolipoprotein B; AU, arbitrary units; AUC, area under the curve; DBP, diastolic blood pressure; DIVAS, Dietary Intervention and vascular function; DVP, digital volume pulse; DVP-RI, DVP-reflection index; DVP-SI, DVP-stiffness index; FMD, flow mediated dilatation; IAUC, incremental AUC; LDI, laser Doppler imaging; NEFA, non-esterified fatty acids; NOx, total nitrite and nitrate concentrations; SBP, systolic blood pressure; sICAM-1, soluble intercellular cell adhesion molecule-1; SNP, sodium nitroprusside; sVCAM-1, soluble vascular cell adhesion molecule-1.

## Supplementary Materials

The following are available online at https://www.mdpi.com/2072-6643/11/9/2044/s1, Table S1: Habitual dietary intakes for the study group as a whole and according to APOE genotype.

## Abbreviations

%TE	Total energy
ANCOVA	Analysis of covariance
ANOVA	Analysis of variance
apo	Apolipoprotein
AU	Arbitrary units
AUC	Area under the curve
BMI	Body mass index
CRP	C-reactive protein
CVD	Cardiovascular disease
DBP	Diastolic blood pressure
DIVAS	Dietary intervention and vascular function
DVP	Digital volume pulse
DVP-RI	DVP reflection index
DVP-SI	DVP stiffness index
FMD	Flow mediated dilatation
HDL-C	High-density lipoprotein cholesterol
HOMA-IR	Quantitative insulin resistance index
IAUC	Incremental AUC
LDI	Laser Doppler imaging
LDL-C	Low-density lipoprotein cholesterol
MUFA	Monounsaturated fatty acids
NEFA	Non-esterified fatty acids
NOx	Sum of nitrite and nitrate concentrations
PUFA	Polyunsaturated fatty acids
rQUICKI	Revised quantitative insulin sensitivity check index
SBP	Systolic blood pressure
SFA	Saturated fatty acids
sICAM-1	Soluble intercellular cell adhesion molecule-1
SNP	Sodium nitroprusside
sVCAM-1	Soluble vascular cell adhesion molecule-1
TAG	Triacylglycerol
TC	Total cholesterol
