# Supplementary material for: Impact of the Apolipoprotein E (epsilon) Genotype on Cardiometabolic Risk Markers and Responsiveness to Acute and Chronic Dietary Fat Manipulation
Source: Nutrients. 2019 Sep 1;11(9):2044. doi: 10.3390/nu11092044 (PMC6770634; doi:10.3390/nu11092044)
Supplement: Supplementary file 1 [file nutrients-11-02044-s001.pdf]

Table S1 Habitual dietary intakes for the study group as a whole and according to *APOE* genotype

	All (n=214)	*E2* carriers (n=30)	*E3/E3* (n=127)	*E4* carriers (n=57)	*P* (genotype)[1]
**Genotype Frequency (%)**	-	14	59	27	
Energy, MJ/d	8.6 ± 0.2	8.6 ± 0.4	8.3 ± 0.2	9.3 ± 0.4	0.410
Total fat, %TE	33.3 ± 0.4	34.5 ± 1.5	32.9 ± 0.5	33.6 ± 0.8	0.706
SFA, %TE	11.9 ± 0.2	12.6 ± 0.8	11.7 ± 0.2	12.0 ± 0.4	0.870
MUFA, %TE	11.8 ± 0.2	11.9 ± 0.5	11.8 ± 0.2	11.8 ± 0.3	0.969
n-6 PUFA, %TE	5.25 ± 0.12	5.39 ± 0.35	5.18 ± 0.15	5.33 ± 0.25	0.851
n-3 PUFA, %TE	0.87 ± 0.03	0.93 ± 0.12	0.85 ± 0.03	0.87 ± 0.06	0.752
Trans fatty acids, %TE	0.95 ± 0.03	1.10 ± 0.08[a]	0.89 ± 0.03[b]	0.99 ± 0.05[ab]	0.031[2]
Dietary cholesterol, mg/d	265 ± 9	290 ± 29	250 ± 11	285 ± 18	0.322
Protein, %TE	16.1 ± 0.2	16.3 ± 0.7	16.1 ± 0.3	16.0 ± 0.4	0.987
Carbohydrate, %TE	49.5 ± 0.5	48.8 ± 1.7	49.6 ± 0.6	49.5 ± 1.0	0.969
Dietary fibre (AOAC), g/d	22.4 ± 0.5	21.5 ± 1.5	22.1 ± 0.6	23.6 ± 1.1	0.410

Values represent mean ± SEM. Determined from a single 4-day weighed food and drink diary completed prior to the first study visit of the Dietary Intervention and Vascular Function (DIVAS) and DIVAS-2 studies; dietary assessments not available for n=2. *E2* carriers = *E2/E2* and *E2/E3; E4* carriers = *E3/E4* and *E4/E4*. *E2/E4* individuals were excluded from the analysis.

[1] Data analysed by univariate general linear model (ANCOVA) adjusted for age, BMI, and gender.

[2] Post-hoc analysis identified differences between genotype groups (identified by different superscript letters [a,b,c] within a row), although these were not significant following Bonferroni correction ($P \geq 0.017$).

Abbreviations: %TE, percentage of total energy; AOAC, Association of Official Analytical Chemists; MUFA, monounsaturated fatty acids; PUFA, polyunsaturated fatty acids; SFA, saturated fatty acids.
